# Non-linear Character of Plasma Amyloid Beta Over the Course of Cognitive Decline in Alzheimer’s Continuum

**DOI:** 10.3389/fnagi.2022.832700

**Published:** 2022-03-23

**Authors:** Feng-Feng Pan, Qi Huang, Ying Wang, Yi-Fan Wang, Yi-Hui Guan, Fang Xie, Qi-Hao Guo

**Affiliations:** ^1^Department of Gerontology, Shanghai Jiao Tong University Affiliated Sixth People’s Hospital, Shanghai, China; ^2^PET Center, Huashan Hospital, Fudan University, Shanghai, China

**Keywords:** Alzheimer’s disease (AD), mild cognitive impairment (MCI), subjective cognitive decline (SCD), plasma amyloid-β, Simoa, amyloid-β PET

## Abstract

Plasma amyloid-β (Aβ) was associated with brain Aβ deposition and Alzheimer’s disease (AD) development. However, changes of plasma Aβ over the course of cognitive decline in the Alzheimer’s continuum remained uncertain. We recruited 449 participants to this study, including normal controls (NC), subjective cognitive decline (SCD), mild cognitive impairment (MCI), AD, and non-AD dementia. All the participants underwent plasma Aβ42, Aβ40, and t-tau measurements with single-molecule array (Simoa) immunoassay and PET scan with 18F-florbetapir amyloid tracer. In the subgroup of Aβ-PET positive, plasma Aβ42 and Aβ42/Aβ40 ratio was significantly lower in AD than NC, SCD and MCI, yet SCD had significantly higher levels of plasma Aβ42 than both NC and MCI. In the diagnostic groups of MCI and dementia, participants with Aβ-PET positive had lower plasma Aβ42 and Aβ42/40 ratio than participants with Aβ-PET negative, and the increasing levels of plasma Aβ42 and Aβ42/40 ratio indicated lower risks of Aβ-PET positive. However, in the participants with SCD, plasma Aβ42 and Aβ40 were higher in the subgroup of Aβ-PET positive than Aβ-PET negative, and the increasing levels of plasma Aβ42 and Aβ40 indicated higher risks of Aβ-PET positive. No significant association was observed between plasma Aβ and Aβ-PET status in normal controls. These findings showed that, in the continuum of AD, plasma Aβ42 had a significantly increasing trend from NC to SCD before decreasing in MCI and AD. Furthermore, the predictive values of plasma Aβ for brain amyloid deposition were inconsistent over the course of cognitive decline.

## Introduction

Deposition of amyloid-β (Aβ) plaques and neurofibrillary tangles (NFTs) of tau fibrils are still the primary neuropathological hallmarks of Alzheimer’s disease (AD) (Long and Holtzman, 2019). Though the examination of cerebrospinal fluid (CSF) and PET imaging provide the most reliable AD-related biomarkers of Aβ and tau *in vivo* (Engelborghs et al., 2008; Brier et al., 2016), the invasive sampling procedure of CSF and high cost of PET image hinder their application in clinical trials and population screening, especially in the preclinical stages of AD. Blood-based biomarkers have the advantages of less invasive, more cost effective and easy to handle, potentially enabling its usage in population screening and follow-up. Owing to the emergence of ultrasensitive technologies such as immunoprecipitation–mass spectrometry (IP–MS) (Kaneko et al., 2014) and single molecule array (Simoa) (Li and Mielke, 2019), measuring AD-related biomarkers in blood becomes increasingly promising. With these high sensitivity measurements, previous studies showed that plasma Aβ was significantly correlated with CSF (Janelidze et al., 2016; Nakamura et al., 2018; Verberk et al., 2018), and had a high performance in predicting cerebral amyloid deposition (Nakamura et al., 2018; Schindler et al., 2019; Vergallo et al., 2019). Consistent with the association between low plasma Aβ and increasing cerebral amyloid deposition, plasma Aβ isoforms and Aβ42/Aβ40 ratio were significantly lower in patients with AD (Rembach et al., 2014; Janelidze et al., 2016; Hanon et al., 2018). Even in the dementia-free populations, lower plasma levels of Aβ42 or Aβ42/Aβ40 ratio are associated with steeper rate of cognitive decline or increased risk of AD and dementia (Chouraki et al., 2015; Giudici et al., 2020; Verberk et al., 2020).

However, even though the significant correlations between low plasma Aβ and AD were identified, confusing findings still remained. For example, in the large sample cohort of Australian Imaging Biomarkers and Lifestyle (AIBL) study, plasma concentrations of Aβ42 and Aβ40 measured by ELISA showed an increasing trend from health controls to mild cognitive impairment (MCI), but decreased from MCI to AD (Lui et al., 2010). Similarly, a population based cross-sectional study found that plasma Aβ42 and Aβ42/Aβ40 ratio in the possible cognitive impairment were higher than both the normal cognition group and probable cognitive impairment group (Wang et al., 2018). A recent longitudinal study showed that plasma Aβ42 levels increased gradually before the stage of MCI and decreased just prior to clinical AD onset (Chen et al., 2019). Taken together, these data suggest a dynamic, non-linear character of plasma amyloid-β over the course of cognitive decline. However, this character of plasma amyloid-β was mostly derived from the patients of MCI and dementia, but not included the preclinical stages of AD, such as subjective cognitive decline (SCD) (Jessen et al., 2014). Furthermore, the status of amyloid deposition in the brain was not sufficiently taken into account when the results were observed.

The aims of this study were to compare plasma Aβ42, Aβ40, and t-tau examined via Simoa immunoassay between the diagnostic groups of normal controls (NC), SCD, MCI, AD dementia and non-AD dementia, respectively in Aβ-PET negative and Aβ-PET positive participants, and to explore the relationships between plasma biomarkers and Aβ-PET status in each diagnostic group with different degrees of cognitive decline.

## Materials and Methods

### Study Participants

In this monocentric retrospective cohort study, a total of 449 individuals were enrolled from Sixth People’s Hospital, Shanghai, China, from January 2019 to June 2021. Participants were aged 40–80 years, educated more than 1 year. Individuals with a history of significant neurologic disease, psychiatric disorders, alcoholism, drug abuse and head trauma were excluded. Routine laboratory tests and cranial MRI scanning were carried out to preclude relevant diseases which may be adversely affecting cognitive function, such as abnormalities in folic acid, vitamin B12, thyroid function, cerebral infarction, subdural hematomas, hydrocephalus, intracranial tumors and infections. All the participants underwent a battery of standardized neuropsychological tests and plasma Aβ42, Aβ40, and t-tau were examined via Simoa immunoassay. All the participants underwent 18F-florbetapir PET scan within 3 months after blood sampling. Written informed consent was obtained from all the participants or their caregivers. The Ethics Committee of Shanghai Jiao Tong University Affiliated Sixth People’s Hospital approved this study.

### Neuropsychological Assessment

General cognitive performance was assessed by Mini-Mental State Examination (MMSE) (Katzman et al., 1988) and Chinese version of Montreal Cognitive Assessment-Basic (MoCA-BC) (Huang et al., 2018). Global functional status was assessed by Everyday Cognition (ECOG) (Farias et al., 2008) and Functional Assessment Questionnaire (FAQ) (Pfeffer et al., 1982). In addition, a battery of standardized neuropsychological tests including six neuropsychological indexes was carried out: Auditory Verbal Learning Test (AVLT) (Zhao et al., 2015) 30-min delayed free recall and AVLT recognition for memory, Boston Naming Test (BNT) (Guo et al., 2006) and Animal Verbal Fluency Test (AFT) (Zhao et al., 2013a) for language, Shape Trail Test Part A and B (STT-A and STT-B) (Zhao et al., 2013b) for attention/executive function.

### Cognitive Groups

Dementia were diagnosed according to the criteria of Diagnostic and Statistical Manual of Mental Disorders, 4th edition-revised (DSM-IV-R) (American Psychiatric Association [APA], 2000). The diagnosis of AD was made by experienced neurologists according to the National Institute on Aging and Alzheimer’s Association (NIA-AA) criteria for probable AD dementia (McKhann et al., 2011) and determined by the positive results of 18F-florbetapir PET scan. Dementia patients or clinically diagnosed AD patients with the negative results of 18F-florbetapir PET scan were classified as non-AD dementia. In the non-dementia participants, diagnosis of MCI was given if the participant met one of the following criteria proposed by Bondi et al. (2014): (1) impaired scores (defined as >1 standard deviation (SD) below the age-corrected normative mean) on two of the six neuropsychological indexes in the same cognitive domain; (2) impaired scores in each of the three cognitive domains; (3) FAQ score ≥9. Participants with self-reported experiences of persistent cognitive decline compared with previously normal status but performed normally on the standardized neuropsychological tests were classified as SCD according to the conceptual framework proposed by the Subjective Cognitive Decline Initiative (SCD-I) working group (Jessen et al., 2014). Normal controls were recruited through advertisements or from caregivers who had no obvious cognitive complaint and evidence of cognitive dysfunction.

### Blood Processing and Measurements of Plasma Aβ42, Aβ40, and t-Tau

Blood samples were centrifuged at 500 × *g* for 5 min at 4°C to collect plasma. Then plasma was immediately aliquoted into ultra-low adsorption tubes (AXYGEN MCT-150-L-C) on ice and stored at −80°C refrigerator. Before the test, plasma samples were transferred from the refrigerator to ice plate for 30 min, and then centrifuged at 10,000 × *g* for 5 min at 4°C. The measurements of plasma Aβ42, Aβ40, and t-tau were performed on the Quanterix Simoa HD-1 platform (Wilson et al., 2016), with Neurology 3-Plex A Assay Kit (Lot 502838). Reagent pretreatment and sample loading were carried out according to the instructions of the manufacture. In brief, the plasma samples were diluted 1:4 according to the minimum required dilution (MRD) and the diluted sample volume per measurement was 152 μL. Concentrations of each plasma biomarker (pg/mL) were calculated from the calibration curve. All the biomarker measurements were performed by laboratory technicians who were blinded to the clinical data.

### 18F-Florbetapir Positron Emission Tomography Acquisition and Analysis

Amyloid PET images were obtained from a PET/CT system (Biograph mCT Flow PET/CT, Siemens, Erlangen, Germany) at the PET center of Huashan hospital, Fudan University. Cerebral amyloid PET scans were carried out 50 min after the intravenous injection of 7.4 MBq/kg (0.2 mCi/kg) florbetapir and lasted for 20 min. PET images were reconstructed using filtered back projection algorithm with corrections for decay, normalization, dead time, photon attenuation, scatter and random coincidences. PET images were coregistered to the individual structural MRI and spatially normalized in the Montreal Neurological Institute (MNI) template. Standard uptake value ratios (SUVRs) were calculated for the cortical regions of interest (ROIs) relative to cerebellar crus, including posterior cingulate, precuneus, temporal, frontal and parietal lobes. Global SUVR scores were calculated by weighted averaging of these ROIs. The positive 18F-florbetapir PET images were defined by the method of visual rating according to the guidelines for interpreting amyloid PET (Lundeen et al., 2018). All the amyloid PET images were judged by three physicians independently and the results were determined if more than two physicians made the same judgment.

### Statistical Analyses

Characteristics of the diagnostic groups were compared by one-way analysis of variance (ANOVA) or Chi-squared analyses based on the data types. Differences of plasma Aβ and t-tau among diagnostic groups along with Aβ-PET status were assessed with general linear models adjusted for age, sex and education years. Logistic regression analysis was used to study the relationships between plasma Aβ and 18F-florbetapir PET results. Odds ratios (ORs) and their 95% confidence intervals (CIs) were calculated to provide an estimation of the magnitude of associations. All the hypothesis testing was two-sided, and the level of significance was set at α = 0.05. Statistical analyses were conducted using IBM SPSS Statistics 23.0. A graphics package (GraphPad Prism, version 8.0) was used to create figures.

## Results

### Demographic and Clinical Characteristics

Demographic and clinical characteristics for the groups of NC, SCD, MCI, AD, and non-AD dementia are shown in Table 1. Normal controls were significantly younger and patients of AD and non-AD dementia had a significantly lower education level. There was no significant difference of gender, diabetes prevalence, hypertension prevalence and hyperlipidemia prevalence among the groups. Compared to the participants with NC and SCD, performances of neuropsychological tests decreased significantly for MCI, AD, and non-AD dementia. Individuals of SCD performed worse than NC on AVLT delayed free recall (*P* = 0.009) and AFT (*P* = 0.001). For the dementia groups, patients of AD performed worse than non-AD dementia on MMSE (*P* < 0.001), AVLT delayed free recall (*P* = 0.030), AVLT recognition (*P* < 0.001), BNT (*P* = 0.045), FAQ (*P* < 0.001) and ECOG (*P* = 0.029). The 18F-florbetapir PET positive prevalence for the groups of NC, SCD, and MCI were 20.8, 35.1, and 38.7%, respectively. All the 18F-florbetapir PET images were positive for individuals of AD and negative for individuals of non-AD dementia.

**TABLE 1:** Demographics, neuropsychological tests and 18F-florbetapir PET imaging for NC, SCD, MCI, AD, and non-AD dementia.

Index	NC (*n* = 183)	SCD (*n* = 77)	MCI (*n* = 111)	AD (*n* = 56)	Non-AD dementia (*n* = 22)	*F*/*x*^2^ (*P*-value)
**Demographics**						
Age (years)	61.99 ± 8.26	66.39 ± 4.54	65.11 ± 7.06	64.46 ± 7.09	64.27 ± 6.22	6.36 (<0.001)
Education (years)	12.75 ± 2.92	12.09 ± 2.91	11.03 ± 3.16	8.49 ± 4.46	8.09 ± 3.22	26.13 (<0.001)
Gender (M:F)	73:110	26:51	38:73	22:34	14:8	7.61 (0.107)
Diabetes [*n* (%)]	27 (14.8%)	13 (16.9%)	13 (11.7%)	7 (12.5%)	5 (22.7%)	2.35 (0.671)
Hypertension [*n* (%)]	62 (33.9%)	21 (27.3%)	33 (29.7%)	11 (19.6%)	10 (45.5%)	6.82 (0.145)
Hyperlipidemia [*n* (%)]	33 (18.0%)	13 (16.9%)	16 (14.4%)	7 (12.5%)	3 (13.6%)	1.41 (0.842)
**Neuropsychological tests**						
MMSE	28.21 ± 1.56	27.90 ± 1.60	26.51 ± 2.00	17.06 ± 5.12	19.90 ± 4.71	237.37 (<0.001)
MoCA-BC	26.12 ± 2.39	25.52 ± 2.30	21.78 ± 3.38	13.06 ± 4.35	13.85 ± 4.68	238.38 (<0.001)
AVLT delayed recall	5.81 ± 2.25	5.05 ± 2.15	2.31 ± 2.07	0.44 ± 0.75	2.09 ± 1.70	75.87 (<0.001)
AVLT recognition	22.10 ± 1.51	21.64 ± 1.80	17.89 ± 2.73	14.84 ± 5.46	18.18 ± 2.08	96.61 (<0.001)
BNT	24.75 ± 2.76	24.10 ± 2.86	21.21 ± 4.01	15.89 ± 6.71	18.45 ± 4.36	51.23 (<0.001)
AFT	17.60 ± 3.99	15.69 ± 4.20	12.75 ± 3.22	9.26 ± 3.59	10.27 ± 2.61	53.13 (<0.001)
STT-A	46.24 ± 14.46	45.48 ± 12.10	59.25 ± 28.65	82.26 ± 53.07	73.91 ± 28.87	20.38 (<0.001)
STT-B	116.77 ± 33.65	127.40 ± 39.93	156.26 ± 50.63	171.17 ± 82.56	166.09 ± 70.62	18.98 (<0.001)
FAQ	0.29 ± 0.91	0.77 ± 1.84	0.89 ± 2.20	6.12 ± 6.95	2.45 ± 2.66	43.17 (<0.001)
ECOG	17.36 ± 5.83	18.33 ± 5.47	20.81 ± 7.90	29.86 ± 9.57	25.43 ± 8.77	34.22 (<0.001)
18F-florbetapir PET positive	38 (20.8%)	27 (35.1%)	43 (38.7%)	56 (100%)	0 (0%)	129.88 (<0.001)

*NC, cognitively normal controls; SCD, subjective cognitive decline; MCI, mild cognitive impairment; AD, Alzheimer’s disease; MMSE, Mini-Mental State Examination; MoCA-BC, Chinese version of Montreal Cognitive Assessment-Basic; AVLT, Auditory Verbal Learning Test; BNT, Boston Naming Test; AFT, Animal Verbal Fluency Test; STT-A and B, Shape Trail Test Part A and B; FAQ, Functional Assessment Questionnaire; ECOG, Everyday Cognition; 18F-florbetapir PET, 18F-florbetapir positron emission tomography.*

### Plasma Aβ42, Aβ40, Aβ42/Aβ40, and t-Tau Among Diagnostic Groups

**FIGURE 1:**
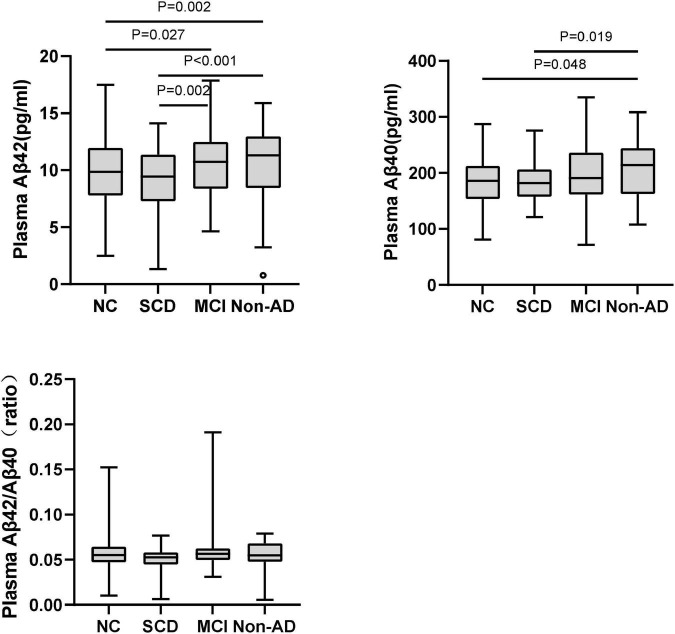
Pairwise comparisons of plasma Aβ42, Aβ40, and Aβ42/Aβ40 among NC, SCD, MCI, and non-AD dementia with Aβ-PET (–). *P*-values were adjusted for age, sex, and education years. The black circles represented the outliers.

**FIGURE 2:**
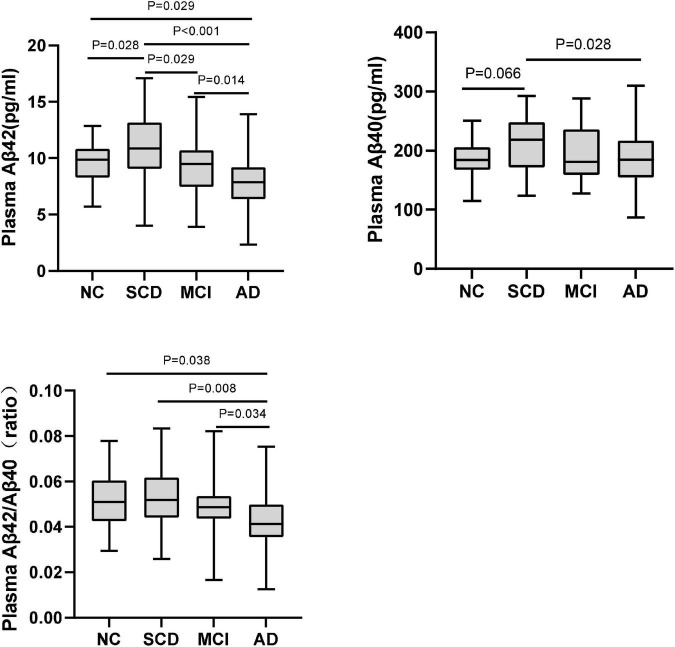
Pairwise comparisons of plasma Aβ42, Aβ40, and Aβ42/Aβ40 among NC, SCD, MCI, and AD dementia with Aβ-PET (+). *P*-values were adjusted for age, sex, and education years.

As shown in Figure 1 and Table 2, in the subgroup of Aβ-PET negative, no significant difference was found for plasma Aβ40, Aβ42/Aβ40 ratio, and t-tau among the diagnostic groups, except for plasma Aβ42 (*P* = 0.001). Pairwise comparisons indicated that plasma Aβ42 was significantly higher in MCI and non-AD dementia than NC and SCD (*P* = 0.027 and *P* = 0.002 for MCI vs. NC and SCD; *P* = 0.002 and *P* < 0.001 for non-AD dementia vs. NC and SCD), while no significant difference was found between NC and SCD, or between MCI and non-AD dementia. Though no statistically significant differences between the diagnostic groups overall, the level of plasma Aβ40 in non-AD was significantly higher than in NC and SCD (*P* = 0.019 and *P* = 0.048, respectively). In this Aβ-PET negative population, no significant difference of 18F-florbetapir PET SUVR was found between the groups of NC, SCD, MCI, and non-AD dementia (Figure 2).

**TABLE 2:** Plasma Aβ42, Aβ40, Aβ42/Aβ40, t-tau, and 18F-florbetapir PET SUVR for NC, SCD, MCI, and non-AD dementia in individuals with Aβ-PET (−).

Plasma biomarkers	NC (*n* = 145)	SCD (*n* = 50)	MCI (*n* = 68)	Non-AD dementia (*n* = 22)	*F* (*P-*value)
Aβ42 (pg/ml)	9.93 ± 3.01	9.30 ± 2.72	10.83 ± 2.89	11.65 ± 4.41	**6.02 (0.001)**
Aβ40 (pg/ml)	183.86 ± 44.15	184.61 ± 36.19	192.59 ± 52.05	209.01 ± 52.52	1.93 (0.124)
Aβ42/Aβ40 (ratio)	0.0557 ± 0.0172	0.0514 ± 0.0139	0.0585 ± 0.0200	0.0555 ± 0.0144	2.10 (0.101)
T-tau (pg/ml)	2.50 ± 1.23	2.29 ± 0.90	2.42 ± 1.00	2.32 ± 0.92	0.27 (0.844)
18F-florbetapir PET SUVR	1.19 ± 0.07	1.19 ± 0.07	1.17 ± 0.08	1.16 ± 0.09	0.18 (0.905)

NC, cognitively normal controls; SCD, subjective cognitive decline; MCI, mild cognitive impairment; AD, Alzheimer’s disease; 18F-florbetapir PET, 18F-florbetapir positron emission tomography; SUVR, standard uptake value ratios. P-values were adjusted for age, sex, and education years with general linear models. The bold values represented the values with statistical significance.

**FIGURE 3:**
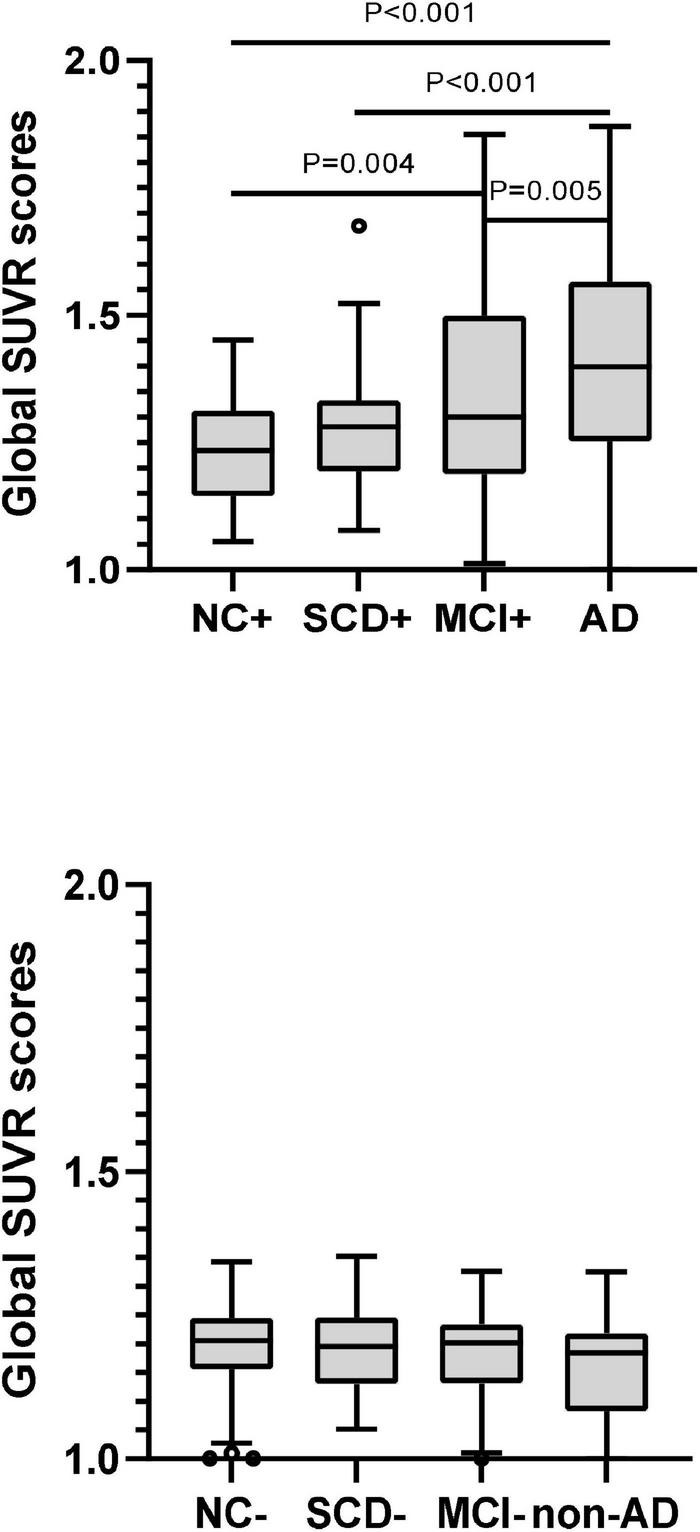
Pairwise comparisons of global SUVR scores over the course of cognitive decline in participants with Aβ-PET (+) and Aβ-PET (–). *P*-values were adjusted for age, sex, and education years. The black circles represented the outliers.

The plasma levels of Aβ42, Aβ40, Aβ42/Aβ40 ratio, and t-tau in different diagnostic groups with Aβ-PET positive were shown in Figure 3 and Table 3. Though the differences of plasma Aβ40 and t-tau were not significant, plasma Aβ42 and Aβ42/Aβ40 ratio showed significant differences among the diagnostic groups (*P* < 0.001 and *P* = 0.032, respectively). Pairwise comparisons indicated that, besides the significantly lower levels of plasma Aβ42 in AD than all other groups, plasma Aβ42 showed significantly higher in SCD than in NC, MCI, and AD (*P* = 0.028, *P* = 0.029, and *P* < 0.001, respectively), yet no significant difference was found between NC and MCI. Though no statistically significant differences between the diagnostic groups overall, the level of plasma Aβ40 in SCD was significantly higher than in AD and marginally higher than in NC (*P* = 0.028 and *P* = 0.066, respectively). The plasma Aβ42/Aβ40 ratio was significantly lower in AD than in NC, SCD, and MCI (*P* = 0.038, *P* = 0.008, and *P* = 0.034, respectively), though no significant difference was found between NC, SCD and MCI. In this Aβ-PET positive population, with the aggravation of cognitive impairment, a significant increasing trend of 18F-florbetapir PET SUVR was observed (*P* < 0.001). Significant difference of 18F-florbetapir PET SUVR was found between the groups of NC and MCI (*P* = 0.004), NC and AD (*P* < 0.001), SCD and AD (*P* < 0.001), MCI and AD (*P* = 0.005) (Figure 2).

**TABLE 3:** Plasma Aβ42, Aβ40, Aβ42/Aβ40, t-tau, and 18F-florbetapir PET SUVR for NC, SCD, MCI, and AD in individuals with Aβ-PET (+).

Plasma biomarkers	NC (*n* = 38)	SCD (*n* = 27)	MCI (*n* = 43)	AD (*n* = 56)	*F* (*P-*value)
Aβ42 (pg/ml)	9.52 ± 1.89	10.83 ± 3.10	9.45 ± 2.58	7.80 ± 2.52	**6.59 (<0.001)**
Aβ40 (pg/ml)	186.47 ± 26.93	211.46 ± 47.05	194.72 ± 43.10	186.41 ± 49.21	1.78 (0.152)
Aβ42/Aβ40 (ratio)	0.0517 ± 0.0110	0.0519 ± 0.0136	0.0493 ± 0.0116	0.0428 ± 0.0129	**3.01 (0.032)**
T-tau (pg/ml)	2.05 ± 0.84	2.39 ± 0.89	2.41 ± 0.99	2.54 ± 1.23	2.46 (0.064)
18F-florbetapir PET SUVR	1.23 ± 0.10	1.28 ± 0.13	1.35 ± 0.22	1.41 ± 0.22	**11.93 (<0.001)**

NC, cognitively normal controls; SCD, subjective cognitive decline; MCI, mild cognitive impairment; AD, Alzheimer’s disease; 18F-florbetapir PET, 18F-florbetapir positron emission tomography; SUVR, standard uptake value ratios. P-values were adjusted for age, sex, and education years with general linear models. The bold values represented the values with statistical significance.

### Associations Between Plasma Amyloid-β and Amyloid-β-Positron Emission Tomography in Different Diagnostic Groups

**FIGURE 4:**
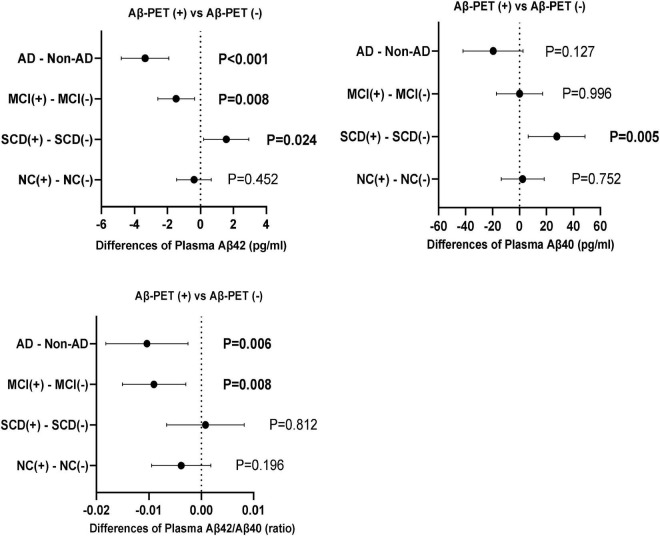
Differences of plasma Aβ42, Aβ40, and Aβ42/Aβ40 between Aβ-PET (+) and Aβ-PET (–) in the diagnostic groups of NC, SCD, MCI and dementia. *P*-values were adjusted for age, sex, and education years.

The comparisons of plasma Aβ42, Aβ40 and Aβ42/Aβ40 between the subgroups of Aβ-PET positive and negative were carried out in different diagnostic groups (Figure 4). In the participants with dementia and MCI, both plasma Aβ42 level and Aβ42/40 ratio were significantly lower in the subgroup of Aβ-PET positive than in Aβ-PET negative (*P* < 0.001 and *P* = 0.006 in dementia, respectively; both *P* = 0.008 in MCI), though no significant difference was found for plasma Aβ40. However, in the participants with SCD, both the levels of plasma Aβ42 and Aβ40 were significantly higher in the subgroup of Aβ-PET positive than Aβ-PET negative (*P* = 0.024 and *P* = 0.005, respectively), though no significant difference was found for plasma Aβ42/Aβ40 ratio. In the participants with normal controls, no significant difference was observed between the subgroups of Aβ-PET positive and Aβ-PET negative for plasma Aβ42, Aβ40 and Aβ42/Aβ40 ratio. Binary logistic regression models adjusted for age, sex and education years were used to assess the relationship between plasma Aβ and results of 18F-florbetapir PET (Table 4). In all the participants, increasing levels of plasma Aβ42 and Aβ42/Aβ40 ratio (*Z*-value) had lower risks of Aβ-PET positive (OR = 0.904, 95% CI = 0.843–0.968, *P* = 0.004 and OR = 0.589, 95% CI = 0.456–0.760, *P* < 0.001, respectively). In the participants with MCI and dementia, increasing levels of plasma Aβ42 and plasma Aβ42/Aβ40 (*Z*-value) also indicated lower risks of Aβ-PET positive and showed more pronounced OR values (OR = 0.843, 95% CI = 0.727–0.978, *P* = 0.024 and OR = 0.369, 95% CI = 0.192–0.708, *P* = 0.003, respectively in MCI; OR = 0.828, 95% CI = 0.710–0.966, *P* = 0.016 and OR = 0.261, 95% CI = 0.116–0.586, *P* = 0.001, respectively in dementia). However, in the participants with SCD, increasing levels of plasma Aβ42 and Aβ40 had higher risks of Aβ-PET positive (OR = 1.308, 95% CI = 1.067–1.602, *P* = 0.010 and OR = 1.025, 95% CI = 1.010–1.041, *P* = 0.001, respectively), though no significant association was found between plasma Aβ42/Aβ40 and Aβ-PET positive. In the participants with normal controls, no significant association was observed between plasma Aβ and Aβ-PET results.

**TABLE 4:** Logistic regression analysis on relationship between plasma biomarkers and AV45 PET positive in different groups.

	Plasma Aβ 42 (continuous variable)		Plasma Aβ 40 (continuous variable)		Plasma Aβ 42/Aβ 40 (*Z-*value)	
Group	OR (95% CI)	*P-*value	OR (95% CI)	*P-*value	OR (95% CI)	*P-*value
All subjects	0.904 (0.843–0.968)	**0.004**	1.002 (0.998–1.007)	0.336	0.589 (0.456–0.760)	**<0.001**
NC	0.969 (0.849–1.107)	0.645	1.003 (0.993–1.013)	0.544	0.776 (0.518–1.164)	0.221
SCD	1.308 (1.067–1.602)	**0.010**	1.025 (1.010–1.041)	**0.001**	1.095 (0.598–2.003)	0.769
MCI	0.843 (0.727–0.978)	**0.024**	1.001 (0.993–1.009)	0.770	0.369 (0.192–0.708)	**0.003**
Dementia	0.828 (0.710–0.966)	**0.016**	0.990 (0.979–1.001)	0.087	0.261 (0.116–0.586)	**0.001**

Adjusted for age, gender, and education years. NC, cognitively normal controls; SCD, subjective cognitive decline; MCI, mild cognitive impairment; Dementia, including AD and non-AD dementia. The bold values represented the values with statistical significance.

## Discussion

The primary objective of this study was to compare the plasma Aβ42, Aβ40, Aβ42/Aβ40 ratio, and t-tau in a cohort of cognitive decline including NC, SCD, MCI, AD, and non-AD dementia along with their status of Aβ-PET. In the subgroup of Aβ-PET negative, less distinct difference of plasma Aβ were found between the groups of NC, SCD, MCI, and non-AD dementia, which was in agreement with no significant difference of 18F-florbetapir PET SUVR between these groups. In the subgroup of Aβ-PET positive, concentration of plasma Aβ42 and ratio of Aβ42/Aβ40 were significantly lower in AD patients when compared to NC, SCD and MCI, while plasma Aβ40 was less distinct, which was consistent with the previous reports (Rembach et al., 2014; Janelidze et al., 2016; Hanon et al., 2018). Plasma t-tau showed no significant difference among the diagnostic groups either in the subgroup of Aβ-PET negative or in the subgroup of Aβ-PET positive, which was in agreement with the findings in the cohort of BioFINDER (Mattsson et al., 2016). However, it is worth noting that, in the subgroup of Aβ-PET positive, levels of plasma Aβ42 and Aβ40 were obviously higher in the diagnostic group of SCD. Furthermore, plasma Aβ42 showed a significantly non-linear character of initially increasing from NC to SCD, and then decreasing from SCD to MCI and AD. To a great extent, this change was similar to the findings that levels of plasma Aβ were more likely to increase in the early stages of cognitive impairment and decrease prior to clinical AD onset (Lui et al., 2010; Wang et al., 2018; Chen et al., 2019). The significance of this study lies in the fact that this non-linear character of plasma Aβ was determined in the continuum of neurodegenerative process with cerebral amyloid deposition, but not simply associated with the decline of cognitive performance.

In our Aβ-PET positive participants, though SCD showed no significant difference of 18F-florbetapir PET SUVR compared to NC and MCI, and MCI had obviously higher 18F-florbetapir PET SUVR than NC, significantly higher plasma Aβ42 and Aβ40 were still found in SCD, and no significant difference of plasma Aβ was found between the groups of NC and MCI, as observed in previous report (Palmqvist et al., 2019b). This may be attributed to the dynamic exchanges of amyloid-β between central nervous system (CNS) and peripheral blood (Roberts et al., 2014), which was consistent with the previous report that amyloid accumulation was faster in pre-MCI than NC, but similar between MCI and NC (Thomas et al., 2020). At this point, whether the initially elevated level of plasma Aβ in SCD represents more soluble forms of amyloid-β in brain and be a potential window period for anti-Aβ immunotherapy should be concerned and need more studies to confirm. On the other hand, though both plasma Aβ42 and Aβ40 were significantly higher in SCD, plasma Aβ42 showed a continued decline from SCD to MCI and AD, yet no significant difference of plasma Aβ40 was found between SCD and MCI, or between MCI and AD. This was consistent with the fact that plasma Aβ42 and Aβ40 had an initially parallel change followed by a consistent decrease for Aβ42 but a flat line for Aβ40 with disease progression (Palmqvist et al., 2019a). At this point, though the relative ratio of Aβ42 and Aβ40 may normalize the pre-analytical variability and eliminate the inter-individual differences for total Aβ concentration (Wiltfang et al., 2007; Willemse et al., 2017), as plasma Aβ42 and Aβ40 increased parallelly in the group of SCD, the change of Aβ42/Aβ40 ratio in this cognitive stage would inevitably be weakened.

In the present study, relationships between the plasma Aβ and results of 18F-florbetapir PET were assessed in our diagnostic groups of NC, SCD, MCI, and dementia, respectively. As a result, though no significant difference of plasma Aβ was observed between the subgroups of Aβ-PET positive and Aβ-PET negative in normal controls, participants of MCI and dementia showed significantly decreased levels of plasma Aβ42 and Aβ42/Aβ40 ratio in the subgroups of Aβ-PET positive when compared to the subgroups of Aβ-PET negative. However, in the participants with SCD, differences of plasma Aβ in the subgroups of Aβ-PET positive versus to Aβ-PET negative were the significantly increased but not decreased levels of plasma Aβ42 and Aβ40. To our knowledge, extracellular Aβ deposits in the brain can be removed by various clearance systems, such as blood–brain barrier (BBB), glymphatic system and meningeal lymphatic vessels (Tarasoff-Conway et al., 2015), which may play a major role in the Aβ clearance from brain to blood (Roberts et al., 2014). Therefore, the inconsistent changes of plasma Aβ between Aβ-PET positive and negative in different diagnostic groups may be associated with the alteration of Aβ clearance in the continuum of AD. For instance, the indifference of plasma Aβ between the normal controls with Aβ-PET positive and Aβ-PET negative may represent an unchanged level of Aβ clearance from brain to blood in this stage. However, the higher level of plasma Aβ in the group of Aβ-PET positive SCD may be attributed to a compensatory increased transportation of Aβ from brain to blood. With disease progression, the significant decreased plasma Aβ42 in MCI and AD may be partly due to the dysfunction of clearance systems in removing Aβ from brain to blood, though it can also be explained by the increased deposition of Aβ42 into plaques (Potter et al., 2013), rather than an intrinsic defect of clearance system. Either way, though it is typically considered that decreased Aβ clearance contribute to the predominant pathogenesis of late-onset AD (LOAD) (Mawuenyega et al., 2010), the elevated plasma Aβ42 and Aβ40 in SCD with Aβ-PET positive indicated that Aβ clearance from brain to blood was not initially impaired, at least in preclinical AD.

Owning to the invasive procedure of CSF examination and high cost of PET image, blood-based assessments with comparable accuracy in predicting cerebral pathology of AD are urgently needed, especially in population screening. However, even previous studies demonstrated that plasma Aβ could be useful as a potential surrogate for brain Aβ pathology, the performances were not sufficient and discrepancies remained (Janelidze et al., 2016; Verberk et al., 2018; Vergallo et al., 2019; De Meyer et al., 2020; Tanaka et al., 2021). In our current study, we explored the predictive values of plasma Aβ for Aβ-PET positive in NC, SCD, MCI, and dementia, respectively. As a result, plasma Aβ had no predictive value for assessing the risk of Aβ-PET positive in normal controls. In the participants with MCI and dementia, increasing levels of plasma Aβ42 and plasma Aβ42/Aβ40 indicated lower risks of Aβ-PET positive, especially for plasma Aβ42/Aβ40 ratio. However, apparently opposing result was obtained in the participants with SCD, which indicated that increasing levels of plasma Aβ42 and Aβ40 had higher risks of Aβ-PET positive, especially for plasma Aβ42. In view of this inconsistent result, we speculate that the insufficient and discrepancy value of plasma Aβ in predicting brain Aβ pathology in previous studies may be due to the different cognitive status of their study populations. We therefore suggest that the validation of plasma Aβ in predicting brain Aβ pathology should be studied in subgroups with different cognitive functions.

Several limitations should be noted in this study. First, designed as a cross sectional study, longitudinal data are needed to confirm the evolution of plasma Aβ in the continuum of AD. Second, factors that might affect the levels of plasma Aβ were not investigated in this study, such as serum albumin, serum creatinine and other parameters related to Aβ clearance, which may raise the possibility of a measurement bias. Third, though the voxel-based analysis of Aβ PET may be more informative to show Aβ accumulated regions associated with elevated plasma Aβ in SCD, it was not available in this study and we will do this in our future direction.

## Conclusion

The present study demonstrated that plasma Aβ42 and Aβ42/Aβ40 ratio were significantly lower in AD patients. In the continuum of AD determined by 18F-florbetapir PET, plasma Aβ42 showed a significantly increasing trend from NC to SCD before decreasing in the groups of MCI and AD. In the participants with MCI and dementia, plasma Aβ42 and Aβ42/Aβ40 ratio were significantly lower in the subgroups of Aβ-PET positive than Aβ-PET negative, and their increasing levels had lower risks of Aβ-PET positive. In contrary, individuals of SCD had increased levels of plasma Aβ42 and Aβ40 in the subgroups of Aβ-PET positive and increasing levels of plasma Aβ42 and Aβ40 indicated higher risks of Aβ-PET positive. Our findings encourage future longitudinal investigations on the changes of plasma Aβ along with cerebral amyloid deposition and cognitive decline, and to further explore the mechanisms of non-linear changes of plasma Aβ with disease progression.

## Data Availability Statement

The raw data supporting the conclusions of this article will be made available by the authors, without undue reservation.

## Ethics Statement

The studies involving human participants were reviewed and approved by the Ethics Committee of Shanghai Jiao Tong University Affiliated Sixth People’s Hospital. The patients/participants provided their written informed consent to participate in this study.

## Author Contributions

FP analyzed and interpreted the data and was a major contributor in writing the manuscript. QH contributed to the PET experiments and drafted and revised the manuscript. YW performed the blood sample collection and processing. YFW had a major role in the acquisition of data. YG had a major role in PET experiments and data analyses. FX assisted in the PET experiments and revised the manuscript. Q-HG designed and conceptualized the study, and revised the manuscript. All authors read and approved the final version of the manuscript.

## Conflict of Interest

The authors declare that the research was conducted in the absence of any commercial or financial relationships that could be construed as a potential conflict of interest.

## Publisher’s Note

All claims expressed in this article are solely those of the authors and do not necessarily represent those of their affiliated organizations, or those of the publisher, the editors and the reviewers. Any product that may be evaluated in this article, or claim that may be made by its manufacturer, is not guaranteed or endorsed by the publisher.
